# The Effect of Statins on Male Reproductive Parameters: A Mechanism Involving Dysregulation of Gonadal Hormone Receptors and TRPV1

**DOI:** 10.3390/ijms24119221

**Published:** 2023-05-25

**Authors:** Temidayo S. Omolaoye, Asha C. Cyril, Rajan Radhakrishnan, Surendra Singh Rawat, Noushad Karuvantevida, Stefan S. du Plessis

**Affiliations:** 1 College of Medicine, Mohammed Bin Rashid University of Medicine and Health Sciences, Dubai P.O. Box 505055, United Arab Emirates; temidayo.omolaoye@mbru.ac.ae (T.S.O.); asha.cyril@mbru.ac.ae (A.C.C.); rajan.radhakrishnan@mbru.ac.ae (R.R.); surendrasingh.rawat@mbru.ac.ae (S.S.R.); noushad.karuvantevida@mbru.ac.ae (N.K.); 2 Division of Medical Physiology, Faculty of Medicine and Health Sciences, Stellenbosch University, Tygerberg, Cape Town 7505, South Africa

**Keywords:** simvastatin, rosuvastatin, male infertility, inflammation, testicular pain, sex hormone receptors

## Abstract

Statins have been shown to cause diverse male reproductive function impairment, and in some cases, orchialgia. Therefore, the current study investigated the possible mechanisms through which statins may alter male reproductive parameters. Thirty adult male Wistar rats (200–250 g) were divided into three groups. The animals were orally administered rosuvastatin (50 mg/kg), simvastatin (50 mg/kg), or 0.5% carboxy methyl cellulose (control), for a 30-day period. Spermatozoa were retrieved from the caudal epididymis for sperm analysis. The testis was used for all biochemical assays and immunofluorescent localization of biomarkers of interest. Rosuvastatin-treated animals presented with a significant decrease in sperm concentration when compared to both the control and simvastatin groups (*p* < 0.005). While no significant difference was observed between the simvastatin and the control group. The Sertoli cells, Leydig cells and whole testicular tissue homogenate expressed transcripts of solute carrier organic anion transporters (*SLCO1B1* and *SLCO1B3*). There was a significant decrease in the testicular protein expression of the luteinizing hormone receptor, follicle stimulating hormone receptor, and transient receptor potential vanilloid 1 in the rosuvastatin and simvastatin-treated animals compared to the control. The expression of *SLCO1B1*, *SLCO1B2*, and *SLCO1B3* in the different spermatogenic cells portray that un-bio transformed statin can be transported into the testicular microenvironment, which can subsequently alter the regulation of the gonadal hormone receptors, dysregulate pain-inflammatory biomarkers, and consequently impair sperm concentration.

## 1. Introduction

Statins (3-hydroxyl 3-methyl glutaryl-Coenzyme A reductase inhibitors) remain the cornerstone therapy for lowering blood lipid levels, and also serve as the main protective factor against cardiovascular diseases [1]. Notwithstanding, there are alarming concerns about its side effects, such as myalgia, muscle atrophy, and in some cases, rhabdomyolysis. Similarly, their effect on male reproductive parameters and function remains controversial; owed to conflicting evidence reported in the literature.

For instance, a study evaluated the effects of differing rosuvastatin doses (3 mg/kg/day or 10 mg/kg/day) on male fertility in Wistar rats. They reported a dose-dependent decrease in sperm motility, sperm concentration, and sperm count [2,3,4]. These animals also displayed abnormal testicular histomorphological structures. These reports are supported by several other authors who showed that upon statin (rosuvastatin, atorvastatin) administration, animals exhibited altered epididymal morphology, experienced delay in ejaculation, and showed reduced fertility potential after natural mating [5,6]. However, several others have reported conflicting results [7].

Adverse effects are also reported in human studies. Such is the study of Pons-Rejraji et al. (2014), which evaluated the effect of atorvastatin (10 mg daily) on 17 young, healthy males for a five-month period. They reported a reduction in total sperm count, and an increase in morphologically abnormal spermatozoa. Atorvastatin also negatively affected the capacitation of sperm up to 3 months after treatment [8]. Furthermore, a case report showed that rosuvastatin administration was able to cause reversible azoospermia in a patient seeking fertility treatment. After 16 weeks of withdrawing from rosuvastatin intake, sperm was seen in the semen, making IVF possible [9]. Not only has the use of different statins caused the aforementioned adverse effects, but they are also shown to cause testicular discomfort, erectile dysfunction, and male sexual dysfunction [10,11,12,13,14]. As reported by Linnebur and Hiatt, a 54-year-old man with hyperlipidemia placed on lovastatin experienced testicular discomfort after 7 months of usage. The patient stopped using lovastatin and was prescribed simvastatin, and later atorvastatin. During treatment period with either simvastatin or atorvastatin, the patient reported feeling testicular pain. This nociception stopped upon cessation of the statin. Although not conclusive, the authors attributed the occurrence of testicular pain to the use of the statin, and further suggested that the adverse effect observed may have been due to the negative effect on testosterone synthesis and secretion [15]. Having highlighted some of the adverse effects of different forms of statin on male reproductive and sexual functions, it is important to highlight the mechanisms through which these negative effects are exerted.

From the current literature, studies have mainly attributed the negative effect observed after statin use to impaired hormonal balance and the subsequent disruption of spermatogenesis, and altered testicular and spermatogenic structures [16,17]. This mechanism is reviewed in detail by Omolaoye et al. [18]. Briefly, during steroidogenesis, the Leydig cell either absorbs cholesterol from the blood via the LDL-receptors or via de novo synthesis. However, upon statin administration, there is reduced circulating LDL-cholesterol, leading to a lesser cholesterol availability in the Leydig cell. Nevertheless, the de novo synthesis is still possible. Studies have shown that upon statin administration, there is a decrease in the LDL-receptors [11,19]; the inability of the LDL-receptors to aid the transport of cholesterol into the Leydig cell has tampered with the bioavailability of cholesterol in these cells, thus making the Leydig cell depend solely on the de novo synthesis of cholesterol. Therefore, the lack of cholesterol bioavailability for testosterone synthesis [12] may subsequently lead to a decrease in testosterone, which may further lead to reduced libido or in some cases, erectile dysfunction.

Although very debatable, findings from both animal and human studies have also proposed that reduced circulating testosterone causes an increase in the generation of pro-inflammatory cytokines, which was reviewed in detail by Mohamad et al. [20]. 

Furthermore, as described by Linnebur and Hiatt, and other authors, statins may cause testicular pain, but the mechanism through which this occurs is unknown [15]. Pain, either acute or chronic, is initiated when the terminal endings of nociceptors are activated by noxious chemicals, or mechanical or thermal stimuli [21]. In neuroscience, it is well evidenced that upon the excitation of nociceptors, the perception of pain ensues [22,23]. Studies have highlighted the role of transient receptor potential vanilloid receptor-1 (TRPV1), purinergic receptor X2 (P2X2), and P2X3 in pain pathogenesis. TRPV1, a ligand-gated, nonselective cation channel has been reported to be expressed not only in dorsal root, trigeminal, and nodose ganglia, but also in non-neuronal tissue and cells [24], including kidney, skin, intestine, heart, and testes [25]. TRPV1 can be activated by ligands (capsaicin, protons, products of arachidonic metabolism, etc.) [25,26,27,28], ambient temperature (37 degrees potentiates its responsiveness to chemical agonist, while temperature above 42 degrees C activates TRPV1 in the absence of exogenous chemical ligand), and chemical ligands [24,29].

Other known pain receptors are the purinergic receptors. Purinergic receptors involved in pain are mostly found in the peripheral terminals where they sense ATP leakage from damaged tissues or released from inflammatory cells [30]. P2X3 is another purinergic receptor that is involved in the pathogenesis of pain. Studies have shown that upon the overexpression of P2X3, there was an increase in the release of inflammatory cytokines and subsequent excitation of nociceptors, which promoted the development of inflammation and the perception of pain [31]. Although their involvement in neural nociception is well evidenced, whether they are activated or involved in driving testicular pain pathogenesis after statin use remains uncertain.

To properly navigate the possibility of statins causing testicular pain, its permeability or transport into the testicular microenvironment should be investigated. During hepatic handling of statins from the blood, statins are transported into the hepatocyte through the solute carrier organic anion transporter (SLC) superfamily, and mainly by the members of the organic anion transporter polypeptides (OATPs). The OATP transporters belong to a superfamily of membrane proteins that mediate the sodium-independent cellular uptake of a variety of amphipathic compounds, such as hormones, bile acids, and many drugs, including statins. The key statins influx transporters in the liver include OATP1B1 and OATP1B3, encoded by *SLCO1B1* and *SLCO1B3*, respectively [32]. Interestingly, studies have shown that these OATPs are present in the testes, and hence, can aid the transport of xenobiotics into the testicular microenvironment. However, whether these genes (*SLCO1B1* and *SLCO1B3*) are present in testes remains uncertain.

Based on this premise, the current study investigated the effect of rosuvastatin (hydrophilic) and simvastatin (lipophilic) on male reproductive parameters, and furthermore evaluated the possible mechanisms through which these effects are exerted. The mechanistic pathways assessed include the pain-related pathway, gonadal hormonal receptor status, and inflammation. Additionally, since statins are transported into the hepatic cells via the OATP1B1 and OATP1B3, and encoded by solute carrier organic anion transporter family member 1B1 (*SLCO1B1*) and *SLCO1B3*, respectively, in humans, we investigated the expression of these genes in the Leydig cells, Sertoli cells, and the whole testes of rats. Their presence may indicate the possibility of residual statin transport into the testicular microenvironment, and thus may consequently affect spermatogenesis.

## 2. Results

### 2.1. Rosuvastatin and Simvastatin on Male Reproductive Parameters

**Figure 1:**
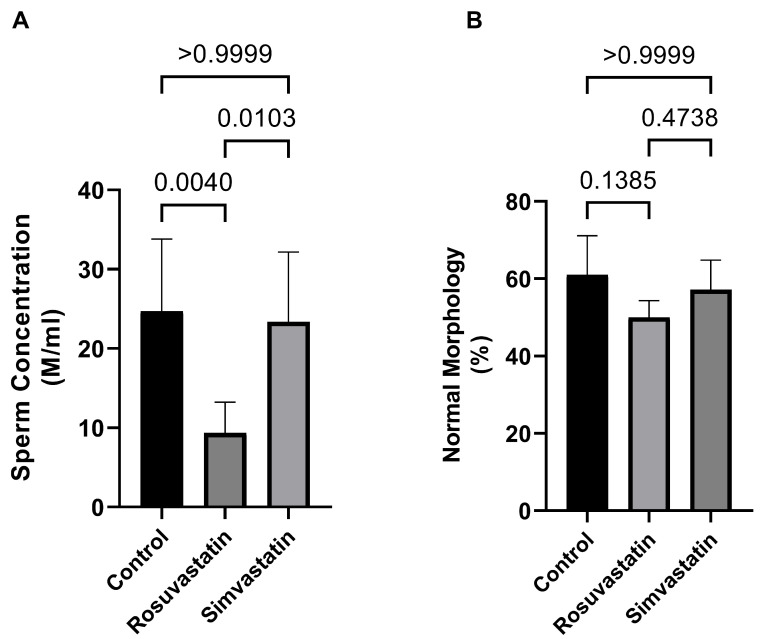
Effects of rosuvastatin and simvastatin on sperm parameters. (**A**) Sperm concentration, *n* = 10; (**B**) Percentage of morphologically normal spermatozoa, *n* = 10. M/mL—million/mL.

The current study evaluated the effect of rosuvastatin and simvastatin administered orally at 50 mg/kgbw on the reproductive parameters of adult male Wistar rats for a 30-day period. In comparison to the control group, rosuvastatin-treated animals presented with a significant decrease in sperm concentration (24.7 ± 9.11 × 10^6^/mL versus 9.36 ± 3.87 × 10^6^/mL; *p* = 0.004), while no change was observed between the simvastatin and control groups (Figure 1A). Nevertheless, there was a significant decrease in the sperm concentration of the rosuvastatin-treated animals compared to the simvastatin group (9.36 ± 3.87 × 10^6^/mL versus 23.37 ± 8.792 × 10^6^/mL; *p* = 0.01, respectively). This raises the question of which form of statin is better, especially in oligozoospermic patients who are seeking infertility treatment. 

In contrast, there was no statistical difference in the percentage of morphologically normal spermatozoa between either the rosuvastatin (50.00 ± 4.36; *p* = 0.13) or simvastatin groups (57.18 ± 7.67; *p* > 0.99) and the control group (61.00 ± 10.15) (Figure 1B).

### 2.2. mRNA Expression of Solute Carrier Transporters (SLCO1B1, SLCO1B2 and SLCO1B3) and Gonadal Hormone Receptors (LHR, FSHR) in Rat Sertoli and Leydig Cells

Since residual statins can be transported into affected cells during redistribution through the OATPs, especially OATP1 and OATP3, which are encoded by *SLCO1B1* and *SLCO1B3*, respectively, in humans; thus, we initially designed primers for these two genes. Using the UCSC genome browser, *SLCO1B1* from the human genome sequence was BLATed, the result spanning region 11,387 on rat genome chromosome 4 with 79.8% was seen. Thereafter, chromosome 4 sequence was blasted on NCBI, and the result showed 100% homology similarities to *SLCO1B2* in the *Rattus norvegicus* (Rat). The rat transcriptomic sequence for *SLCO1B2* was thereafter used to pick primers for *SLCO1B2*. Hence, the mRNA expression levels of these three genes (*SLCO1B1*, *SLCO1B2*, and *SLCO1B3*) were measured in the testicular cells. Additionally, the transcript levels of the luteinizing hormone receptor (*LHR*) and the follicle stimulating hormone receptor (*FSHR*) were also determined in the Leydig and Sertoli cells isolated from rats.

**Figure 2:**
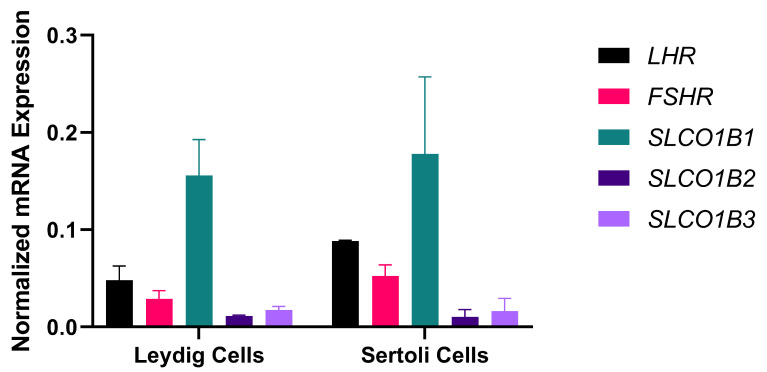
Relative mRNA expression of gonadal hormone receptors (*LHR*, *FSHR*) and solute carrier transporters (*SLCO1B1*, *SLCO1B2* and *SLCO1B3)* in rat Leydig and Sertoli cells. The figure confirms that two of the most important cells (Sertoli and Leydi cells) involved in spermatogenesis maintenance and steroidogenesis, respectively, express LHR, FSHR, and different solute carrier organic anion transporters. These findings enunciate the possibility of their activation and involvement in diverse conditions upon appropriate stimulation. It is important to note that these are baseline values showing the presence of these genes in these cells. Values are representative of a minimum of three biological replicates.

Upon isolation of the cells using the trypan blue method, the concentration of Leydig cell/mL was 2.6 × 10^7^, with 84.6% viability. The concentration of Sertoli cell/mL was 4.04 × 10^6^, with 4.9% viability. Setting qPCR at 40 cycles, the Leydig cell mRNA expression for *SLCO1B1* ranged from 27.125 to 28.548; *SLCO1B2* (30.070–32.437) and *SLCO1B3* ranged from 28.100 to 30.757; while GAPDH ranged from 23.698 to 26.038. The Sertoli cell mRNA expression of *SLCO1B1* was seen at ct range of 25.597–29.445, *SLCO1B2* ranged from 28.613 to 31.726, while the ct value for *SLCO1B3* ranged from 29.970 to 32.501 (values are representative of a minimum of three biological replicates). The ct value for both human *GAPDH* and rat *GAPDH* ranged from 22.763 to 26.409. Additionally, both Leydig and Sertoli cells were confirmed to express transcripts of *LHR* and *FSHR* (Figure 2).

### 2.3. Rosuvastatin and Simvastatin Effect on Testis mRNA Expression of SLCO1B1, SLCO1B2 and SLCO1B3

**Figure 3:**
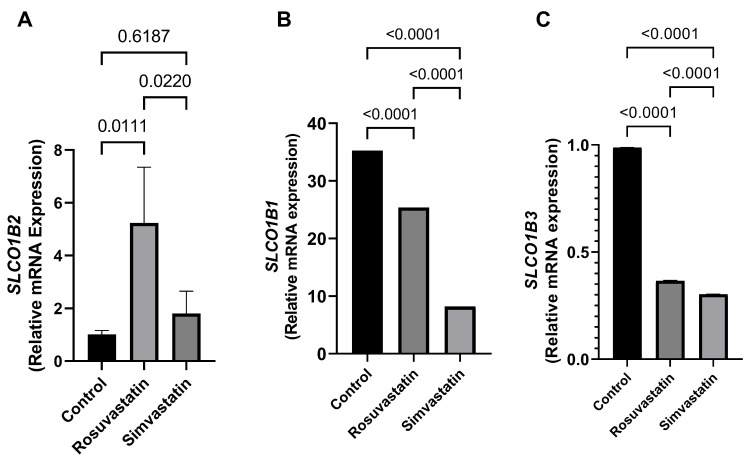
The effect of rosuvastatin and simvastatin on testis mRNA expression of *SLCO1B1*, *SLCO1B2*, and *SLCO1B3*. (**A**) Relative mRNA of *SLCO1B2* in the testes. (**B**) Relative mRNA of SLCO1B1 in the testes. (**C**) Relative mRNA of SLCO1B3 in the testes. SLCO1B1—solute carrier organic anion transporter superfamily 1B1; SLCO1B2—solute carrier organic anion transporter superfamily 1B2; SLCO1B3—solute carrier organic anion transporter superfamily 1B3. Values are representative of a minimum of three biological replicates (*n* = 3–6).

In comparison to the control, the testicular mRNA of *SLCO1B2* was significantly increased (*p* < 0.05) in the rosuvastatin group (1.017 ± 0.1466 versus 5.234 ± 2.114; *p* = 0.01). An increase in trend (77%) was observed in the simvastatin group compared to the control (Figure 3A). In contrast, the mRNA expression of *SLCO1B1* and *SLCO1B3* significantly reduced in the rosuvastatin and simvastatin groups compared to the control group (*p* < 0.0001) (Figure 3B,C).

### 2.4. Rosuvastatin and Simvastatin Effect on Protein Expression of Gonadal Hormone Receptors

One of the most concerning opinions about statin-associated symptoms is the possibility of negatively affecting sex hormones and their receptors, and the resultant adverse consequences on male reproductive health. In lieu, the status of gonadal hormone receptors (LHR and FSHR) was evaluated after rosuvastatin and simvastatin administration.

**Figure 4:**
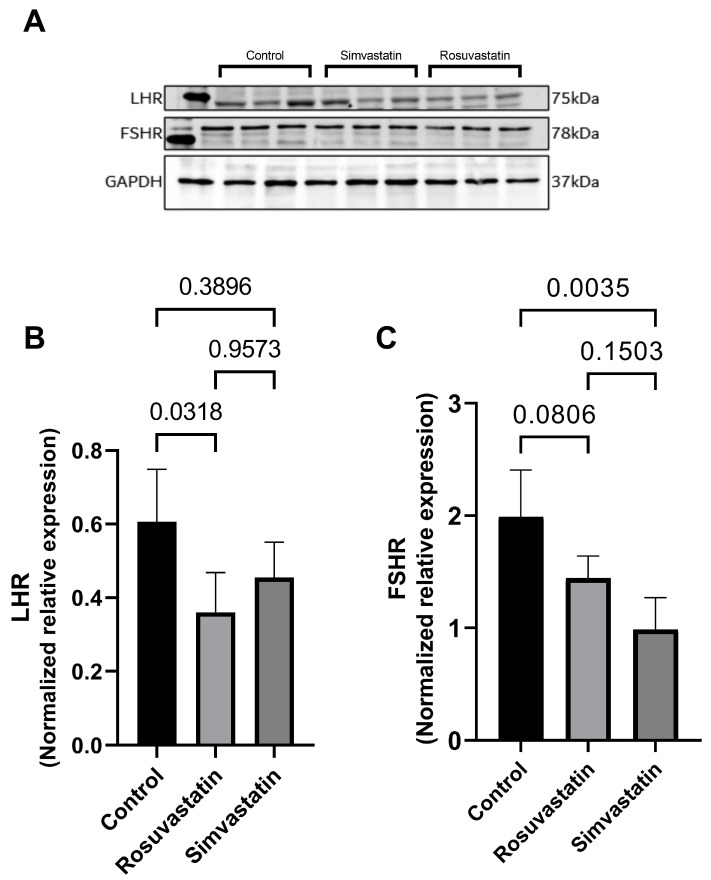
Effect of rosuvastatin and simvastatin on testicular LH and FSH receptors. (**A**) Western blot images of LHR and FSHR. (**B**) Normalized expression of LHR. (**C**) Normalized expression of FSHR. The first lane in the immunoblots is the ladder (molecular weight), while the remaining lanes represent samples from control (*n* = 3), simvastatin (*n* = 3), and rosuvastatin (*n* = 3). Overall, the number of samples reported per group on the graphs include a minimum of six. LHR = Luteinizing hormone receptor; FSHR = follicle stimulating hormone receptor.

There was a significant reduction in the testicular protein expression of LHR in the rosuvastatin-treated animals compared to the control (0.3605 ± 0.1075 versus 0.6063 ± 0.1426; *p* = 0.03 respectively). However, there were no significant changes observed in the testicular LHR expression in simvastatin-treated animals when compared to the control (0.4553 ± 0.0961 versus 0.6063 ± 0.1426; *p* = 0.38 respectively) (Figure 4A,B).

Nevertheless, there was only a trend decrease in the testicular protein expression of FSHR in the rosuvastatin group compared to the control (−27%; *p* = 0.08), while a significant decrease was observed in the simvastatin group compared to the control (*p* < 0.05) (Figure 4A,C).

### 2.5. The Effect of Rosuvastatin and Simvastatin on Pain Receptors (TRPV1, P2X1, P2X3)

Since testicular pain is one of the reported symptoms seen after statin administration, the current study measured the mRNA and protein expression levels of pain-related receptors.

### 2.6. The Effect of Rosuvastatin and Simvastatin on Testicular mRNA Expression of TRPV1, P2X1, P2X3

**Figure 5:**
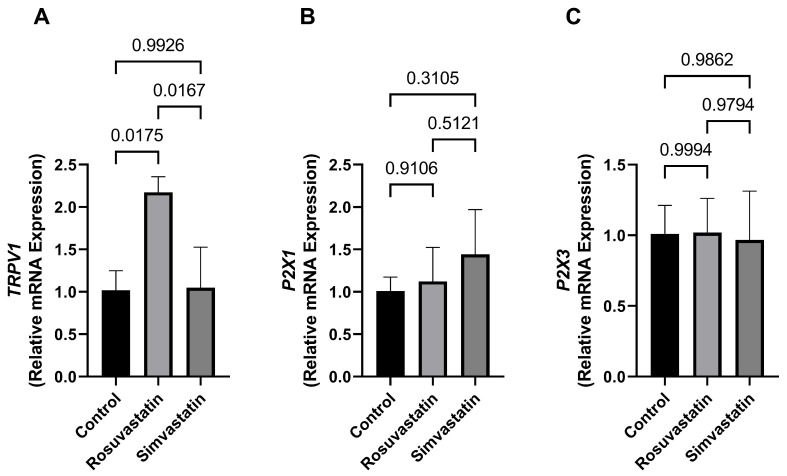
The effect of rosuvastatin and simvastatin on the testicular mRNA expression of pain receptors. (**A**) Relative testicular mRNA expression of *TRPV1*. (**B**) Relative testicular mRNA expression of *P2X1*. (**C**) Relative testicular mRNA expression of *P2X3*. *TRPV1*—transient receptor potential vanilloid 1 gene; *P2X1*—purinergic receptor 1 gene; *P2X3*—purinergic receptor 3 gene. Values are representative of a minimum of four biological replicates (*n* = 4–6).

There was a significant increase in the mRNA expression of *TRPV1* in the rosuvastatin-treated group compared to the control (2.173 ± 0.1836 versus 1.019 ± 0.2299; *p* = 0.01 respectively) and the simvastatin groups (2.173 ± 0.1836 versus 1.048 ± 0.4779; *p* < 0.05). While no change was observed in the simvastatin group compared to the control. Additionally, there were no significant differences in the mRNA expression of *P2X1* and *P2X3* between the treatment groups compared to the control (*p* > 0.05) (Figure 5A–C).

### 2.7. The Effect of Rosuvastatin and Simvastatin on Testicular Protein Expression of TRPV1, P2X1, P2X3

**Figure 6:**
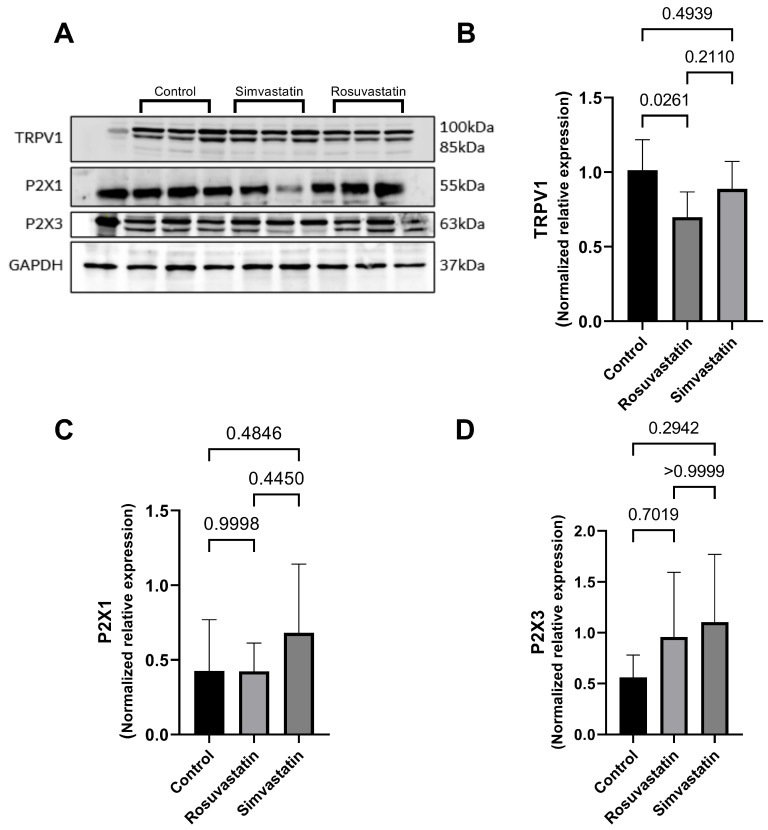
The effect of rosuvastatin and simvastatin on the testicular protein expression of pain receptors. (**A**) Western Blot images of TRPV1, P2X2 and P2X3. (**B**) Testicular protein expression of TRPV1. (**C**) Testicular protein expression of P2X1. (**D**) Testicular protein expression of P2X3. The first lane in the immunoblots is the ladder, while the remaining lanes represent samples from control (*n* = 3), simvastatin (*n* = 3), and rosuvastatin (*n* = 3). Overall, the number of samples reported per group on the graphs include a minimum of six biological replicates.

Animals in the rosuvastatin group displayed a significant decrease in the testicular protein expression of TRPV1 when compared to the control (*p* < 0.05), while a non-significant decline was observed in the simvastatin group compared to the control (0.888 ± 0.183 versus 1.013 ± 0.204), respectively (Figure 6A,B). Furthermore, there was no significant difference in the protein expression of P2X1 and P2X3 between the treatment groups and the control (*p* > 0.05). However, there seems to be an increase in trend in P2X3 protein expression in the treatment groups compared to control (Figure 6A,C,D).

### 2.8. Rosuvastatin and Simvastatin on the Testicular Protein and mRNA Expression of Inflammatory Cytokines

**Figure 7:**
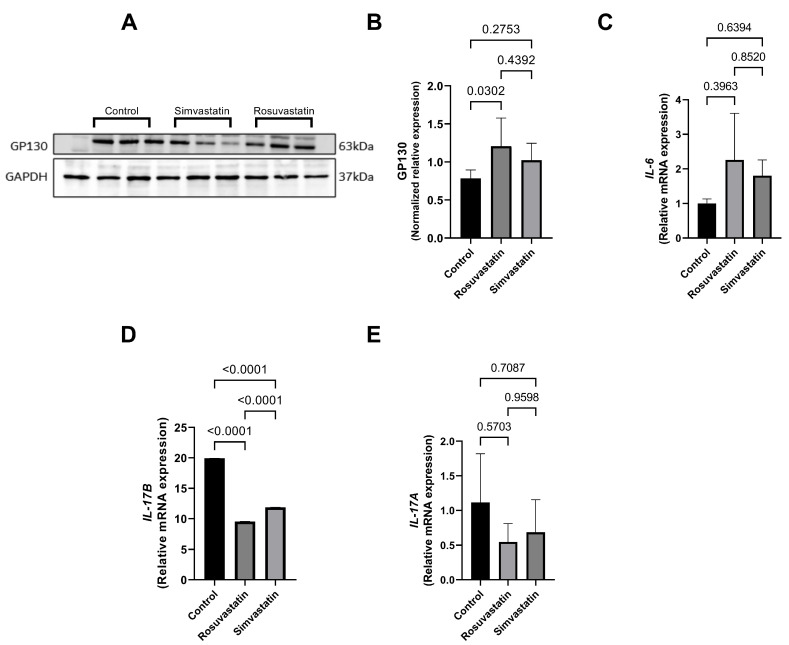
The effect of rosuvastatin and simvastatin on the expression of inflammatory cytokines. (**A**) immunoblot of GP130. (**B**) The protein expression of GP130 in the testes. (**C**) The mRNA expression of *IL-6* in the testes. (**D**) The mRNA expression of testicular *IL-17B*. (**E**) The mRNA expression of testicular *IL-17A*. The first lane in the immunoblots is the ladder, while the remaining lanes represent samples from control (*n* = 3), simvastatin (*n* = 3), and rosuvastatin (*n* = 3). Overall, the number of samples reported per group in the graphs include a minimum of six. For the transcript expression, values are representative of four to six biological replicates.

The association between nociception and inflammation has been recently described. Hence, the current study investigated this relationship by measuring inflammatory cytokines upon statin use, since testicular pain is one of the statin-related symptoms. Following rosuvastatin and simvastatin treatment, there was a significant increase in the protein expression level of GP130 in the rosuvastatin group compared to the control (*p* < 0.05) (Figure 7A,B), while no significance was seen in the simvastatin group compared to the control, although an increase in trend was observed (1.022 ± 0.224 versus 0.784 ± 0.111). 

This increase in trend was also observed in the mRNA expression of *IL-6* in the rosuvastatin (2.262 ± 1.347) and simvastatin (1.806 ± 0.4518) groups compared to the control group (1.004 ± 0.1278) (Figure 7C). Conversely, there was a significant decrease in the mRNA expression of *IL-17B* in the rosuvastatin and simvastatin-treated groups compared to the control (*p* < 0.0001) (Figure 7D), while no significant change was observed in the mRNA expression of *IL-17A* between the groups (Figure 7E).

### 2.9. Qualitative Findings

Using immunofluorescence, we investigated the status and localization of biomarkers of interest in the testes qualitatively.

**Figure 8:**
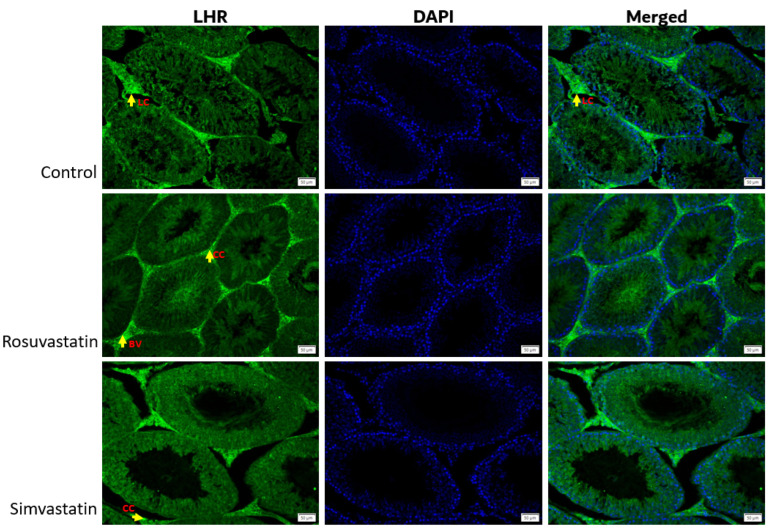
The expression of LHR in the testes. As shown in the figure, LHR is mostly localized in the testicular interstitial, as the different cells in this environment have positive immunoreactivity to LHR. Fluorescence green = immunoreactivity of the protein; DAPI = a counter stain (stains nucleus blue); BV = Blood vessel; CC = connective cell or fibroblast; LC = Leydig cell. Scale bar—50 µm, Magnification—400×.

**Figure 9:**
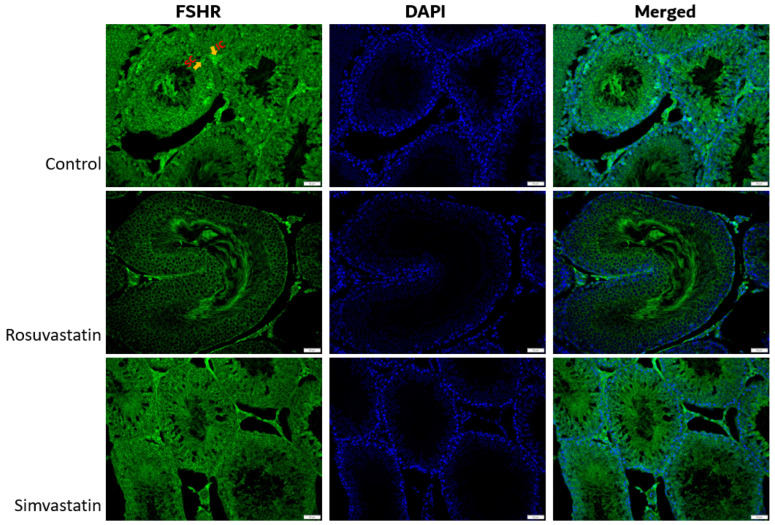
The expression of FSHR in the testes. The Sertoli and interstitial cells express FSHR and there is more positive immunoreactivity to FSHR in the control group compared to both rosuvastatin and simvastatin. Fluorescence green = immunoreactivity of the protein; DAPI = a counter stain (stains nucleus blue); SC = Sertoli cell; IC = Interstitial cells. Scale bar—50 µm, Magnification—400×.

**Figure 10:**
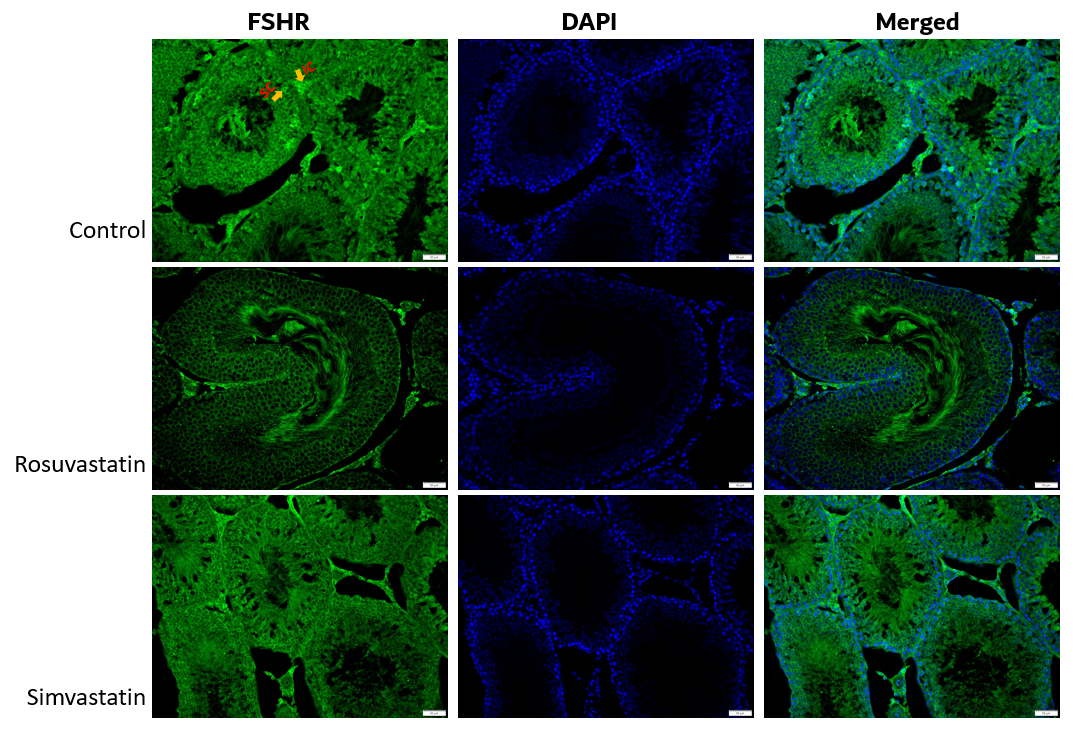
The expression of TRPV1 in the testes. The interstitial cells and primary spermatocytes are immunoreactive to TRPV1. Fluorescence green = immunoreactivity of the protein; DAPI = a counter stain (stains nucleus blue); IC = Interstitial or stroma cells; PS = Primary spermatocyte. Scale bar—50 µm, Magnification—400×.

**Figure 11:**
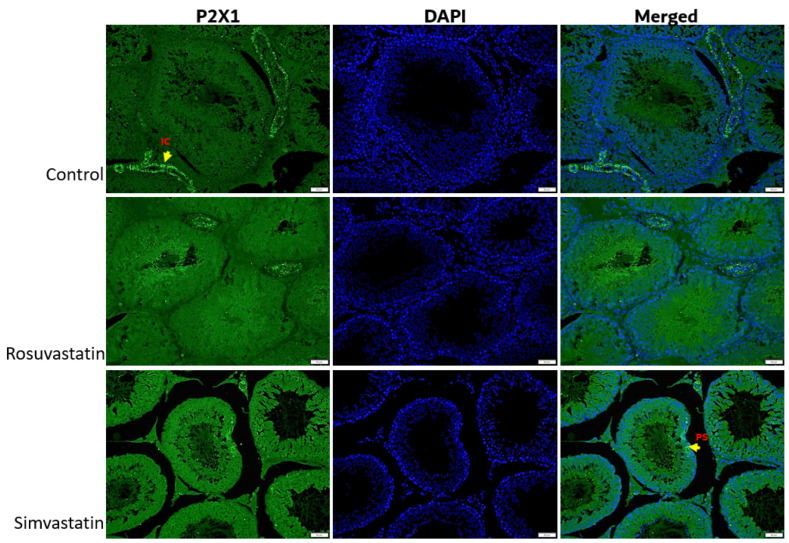
The expression of P2X1 in the testes. P2X1 is expressed in primary spermatocytes and the interstitial cells of the testes. No observable differences are seen in fluorescent intensity between the three groups. Fluorescence green = immunoreactivity of the protein; DAPI = a counter stain (stains nucleus blue); IC = Interstitial cells, PS = Primary spermatocyte. Scale bar—50 µm, Magnification—400×.

**Figure 12:**
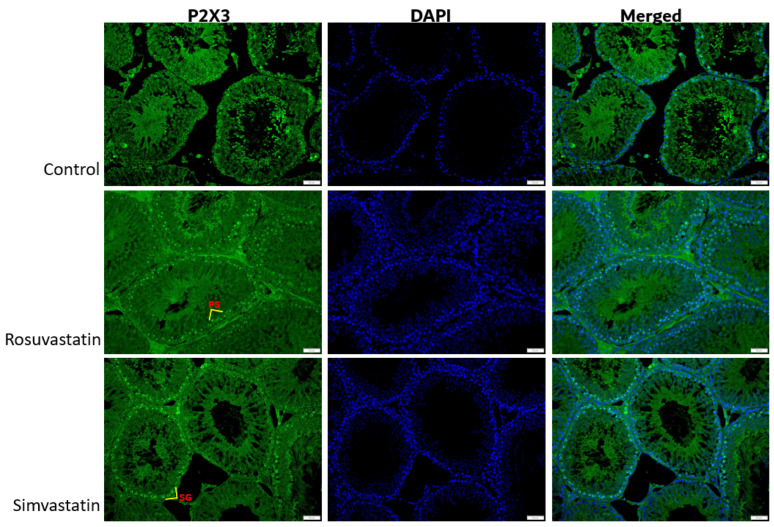
The expression of P2X3 in the testes. P2X3 is expressed in the spermatogonia and primary spermatocytes of the testes. Spermatogonia and primary spermatocytes of both rosuvastatin and simvastatin groups displayed more positive immunoreactivity to P2X3 compared to the control group. Fluorescence green = immunoreactivity of the protein; DAPI = a counter stain (stains nucleus blue); PS = Primary spermatocyte, SG = Spermatogonia, Scale bar—50 µm, Magnification—400×.

**Figure 13:**
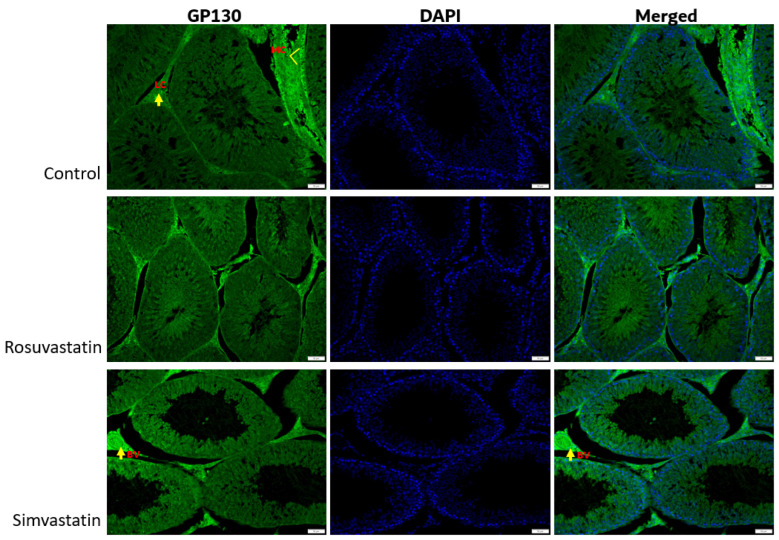
The expression of GP130 in the testes. Myoid cells, Leydig cells and blood vessels show immunoreactivity to GP130. Fluorescence green = immunoreactivity of the protein; DAPI = a counter stain (stains nucleus blue); MC = Myoid cells, LC = Leydig cell, BV = Blood vessel, Scale bar—50 µm, Magnification—400×.

As shown in Figure 8, the Leydig cells showed reactivity to LHR, while the spermatogenic cells showed no reactivity (Figure 8). Sertoli cells and interstitial cells showed immunoreactivity to FSHR (Figure 9). The testicular stroma cells showed positive reactivity to TRPV1 in all groups (Figure 10). The stroma cells and primary spermatocytes of all groups showed less positive reactivity to P2X1 (Figure 11), while the primary spermatocytes of rosuvastatin and simvastatin-treated animals showed greater reactivity to P2X3 (Figure 12). The testicular stroma of all the groups showed reactivity to GP130, especially the blood vessels, and the fibroblast. However, the spermatogenic cells did not show any reactivity (Figure 13).

## 3. Discussion

Although statins are effective in controlling hypercholesterolemia, several statin-associated symptoms have been described, including testicular discomfort, erectile dysfunction, altered semen parameters, and modified steroid hormones production. These adverse effects on male fertility are not generally agreed upon; thus, the impacts of statin use on male fertility are debatable and controversial. To this end, we investigated the effects of two types of statin (rosuvastatin and simvastatin) on male reproductive parameters from a holistic perspective using an animal model.

In the current study, animals treated with hydrophilic rosuvastatin displayed reduced sperm concentration (oligozoospermia), while the simvastatin group showed no difference in comparison to the control group. The former is in agreement with the study of Leite et al. who reported a decrease in sperm production upon administering rosuvastatin of varying dose (5 mg to 40 mg/kgbw) to adult male rats [4]. This effect is also found in humans. Tada et al. reported that upon administration of rosuvastatin at 2.5 mg/day to hypercholesterolemic males, there was a decrease in sperm concentration and total sperm count [9]. The finding of the current study showed no change in sperm concentration after simvastatin treatment, in contrast to those of Shalaby et al., who observed an increase in sperm concentration upon treating hypercholesterolemic rats with simvastatin at 40 mg/kgbw [33]. Although the sperm concentration of animals treated with simvastatin in the current study did not increase in comparison to the control as shown by Shalaby et al., the sperm concentration did not change, but rather decreased percentage wise. The difference between our study and that of Shalaby et al. is that the current study evaluated simvastatin in normal rats, while Shalaby et al. evaluated simvastatin on hypercholesterolemic rats.

Meanwhile, there was no change in the percentage of morphologically normal spermatozoa after treatment with either rosuvastatin or simvastatin, although a percentage decrease was observed. In contrast to our findings, in their controlled trial, Al-Hilli et al. showed that simvastatin improved sperm morphology in infertile patients, but made no mention of its effect on normozoospermia/fertile patients [34]. Similarly, another study reported that there were no significant changes in the sperm quality of hypercholesterolemic men treated with simvastatin at 40 mg for 18 weeks [35]. Although the findings of the current study showed no alteration in sperm morphology upon treatment with simvastatin, further studies are required.

In their study, Silva et al. showed that treatment of normal rats with rosuvastatin (5 mg/kgbw) led to impaired epididymal morphology and reduced frequency of ejaculation, but did not report effects on sperm morphology [5]. Additionally, in the series of reports published by the Leite et al. group, they showed that treatment with rosuvastatin impaired sperm quality [3,32]. Although the findings of the current study support the negative effect of rosuvastatin on sperm concentration, the percentage decrease (−18%) in morphologically normal spermatozoa in these animals did not reach significance. Several other studies have shown that there is a tendency for impaired sperm quality (motility, concentration morphology, sperm count), altered testicular and epididymal morphology, and other impairments such as male sexual dysfunction, orchialgia, or even inflammation following statin use.

Since sperm concentrations were negatively affected in the current study, and several other studies have also shown the adverse influence of statins on sperm parameters, the plausibility of statin influx into the testicular microenvironment was investigated by measuring the transcript expression of the genes that encode statin transporters during hepatic influx.

During hepatic handling of the already absorbed statins from the blood, statins are transported into the hepatocyte through the SLC superfamily, and mainly by the members of the OATPs. The OATP transporters belong to a superfamily of membrane proteins that mediate the sodium-independent cellular uptake of a variety of amphipathic compounds such as hormones, bile acids, and many drugs, including statins. The key statins influx transporters in the liver include OATP1B1 and OATP1B3, encoded by *SLCO1B1* and *SLCO1B3*, respectively [36]. Others include OATP2B1, OATP1A2, and NTCP. The functionalities of these transporters in drug disposition, metabolism and hepatic handling, and their localization in the kidney, brain, and intestine are well documented. However, evidence is lacking when it comes to their role, localization, protein, and transcript expression levels in drug disposition in the testes. In lieu, Hau et al. identified different xenobiotic transporters on the blood-testis-barrier and other testicular cells. It was shown that OATP1A2, OATP1B1, OATP1B3, OATP2A1, OATP2B1, and OATP3A1-V2 are present on the Sertoli cell basal membrane [37]. They further suggested that the location of these transporters may enhance Sertoli cells to play a role in the uptake of endogenous chemicals and drugs. OATP1B1, OATP1B3, and OATP2B1 were also found in the adluminal area of the seminiferous tubules. This suggests the possibility of bidirectional transport of substances between the Sertoli cells and the germ cells. In addition to the presence of these transporters (OATP1B1 and OATP1B3) in the testes, studies have also quantified their transcript expression level in the Sertoli cells [38,39,40,41]. However, these studies did not quantify the transcript level of the genes that encode these protein transporters.

Hence, the current study measured the mRNA expression of the genes that encode OATP1B1 and OATP1B3 in the Sertoli cells, Leydig cells, and whole testis homogenate. It is worth nothing that *SLCO1B1* and *SLCO1B3* transcriptomic sequences used to design the primers were from *homo sapiens*. However, during verification steps, it was found that *SLCO1B1* has 100% homology to *SLCO1B2* of the rat (*Rattus norvegicus*) transcriptomic sequences. Therefore, *SLCO1B1*, *SLCO1B2*, and *SLCO1B3* were measured in these different testicular isolated primary cells (Sertoli and Leydig cells). Our results showed that *SLCO1B2* is expressed in these cells as is *SLCO1B1* and *SLCO1B3*. In the testis, the mRNA expression of *SLCO1B2* significantly increased in the rosuvastatin group, while there was only a trend increase in the simvastatin-treated animals. Although to the best of our knowledge, there are no available studies showing the mRNA status of these genes in the testes following treatment with either rosuvastatin or simvastatin. Nevertheless, Zaher et al., who measured *SLCO1B2* using human transcriptomic sequences, showed that upon targeted knockout of *SLCO1B2* in mice, there was an increased accumulation of statin (pravastatin) in the blood [42], indicating the essential role of *SLCO1B2* in the transport of statin. Additionally, De Gorter et al. reported that upon OATP1B2/*SLCO1B2* knockout in mice, there were reduced liver-to-plasma ratios for atorvastatin and rosuvastatin, but not for simvastatin. Does this mean simvastatin is able to utilize other SLCs transporters? Hau et al. showed that upon *SLCO1B2* knockout in mice, there was a compensatory change in the expression of hepatic OATP1A1, OATP1A4, and SLCO2B1. The reports of Hau et al. implanted the idea that the increase in the transcript expression of *SLCO1B2* found in the rosuvastatin and simvastatin-treated groups in the current study may be a compensatory response to the reduction in *SLCO1B1* and *SLCO1B3.*

Findings from the current study showed a significant reduction in the mRNA expression of *SLCO1B1* and *SLCO1B3* in the rosuvastatin and simvastatin groups. Studies have shown that when the activities of these transporters are reduced, then there is a possible influx of excess statins into other cells. Because the current study did not measure the activity of these transporters, we cannot infer that the reduced expression of *SLCO1B1* and *SLCO1B3* depicts decreased activity. However, it can be speculated that, (i) there is a compensatory response in *SLCO1B2* following a reduction in the expression of *SLCO1B1* and *SLCO1B3*; (ii) it is plausible that rosuvastatin and simvastatin influx into the testicular microenvironment. These hypotheses require further validation.

Taken that these transporters are present in the testicular microenvironment, especially on the BTB [37], then any residual statin can be redistributed into this tissue. In the current study, we evaluated whether the administration of rosuvastatin and simvastatin would have any effect on the protein expression of gonadal hormone (LH and FSH) receptors.

LHR and FSHR are the target receptors for the anterior pituitary-derived hormones, LH and FSH, respectively. Upon posterior hypothalamus stimulation to release GnRH, and the subsequent stimulatory effect of GnRH on the pituitary, there is consequential release of LH and FSH. LH binds its receptor (LHR) on the Leydig cell to produce testosterone and enhance steroidogenesis. Meanwhile, FSH binds its receptor (FSHR) on the Sertoli cell to regulate spermatogonial stem cell differentiation and maintain spermatogenesis. Several studies have reported altered steroidogenesis upon mal-expression of LHR, while several others reported incomplete spermatogenesis when FSHR was knocked out or mal-expressed [43,44,45,46]. For example, a dose-dependent decrease in LH and LHR was reported upon exposure to gamma irradiation which subsequently led to altered steroidogenesis [43]. Another study showed that treatment with LHR agonist increased the level of testosterone and further aided improved steroidogenesis [45]. These reports indicate that LHR is necessary for proper steroidogenesis and normal male reproductive function.

Findings from the current study showed that treatment with rosuvastatin led to a decrease in the protein expression of LHR, while it remained unchanged upon simvastatin treatment. However, FSHR was significantly reduced after simvastatin administration. This is partly in line with reports of Leite et al., who showed that upon treating rats with varying doses of rosuvastatin, there was a decrease in the level of serum LH, FSH, and testosterone concentrations, as well as a decrease in the expression of testicular androgen receptors [3]. Although different proteins were measured, in that the current study evaluated the receptors while Leite et al. evaluated the hormones, nevertheless, studies have shown that upon a dose-dependent decrease in LH, there is an equal response of decrease in LHR. Conversely, upon treating hypercholesterolemic rats with simvastatin, Shalaby et al. reported no change in serum levels of LH, FSH, or testosterone [33]. Meanwhile, LH and FSH were reported to be greatly reduced upon long-term administration of simvastatin in a cohort of young males. This suggests that long-term treatment with simvastatin can alter gonadal hormones and their receptors [47]. Although, testosterone levels were not measured in the current study, it is well evidenced that a decrease in LH and FSH and their respective receptors can alter steroidogenesis, and subsequently result in reduced testosterone production [46,47]. A decrease in testosterone is associated with pathologies, such as altered male sexual health, impaired testicular function, and disrupted spermatogenesis [16,17]. Interestingly, studies have proposed that reduced circulating testosterone is associated with an increase in the generation of pro-inflammatory cytokines. In brief, in an animal model of hypogonadism, reduced testosterone levels were associated to elevated inflammatory cytokines such as IL-6, IL-1β, and TNF-α. After testosterone treatment, only IL-6 was reduced [48,49]. Similarly, findings from human studies showed increased inflammatory cytokines in hypogonadism [50,51]. This means that impaired steroidogenesis that occurs due to reduced LH or LHR can aid the initiation of pro-inflammatory cytokines recruitment, which will later result in inflammation upon the prolonged pathological condition.

Inflammation and pain have been described to have bidirectional association [24,31]. However, discussions about whether statin use can cause any kind of pain, including testicular pain, are underway. Such are the findings of Linnebur and Hiatt, who showed that different statin administration caused testicular discomfort and later testicular pain in a 54-year-old man with hyperlipidemia [15]. This nociception stopped upon cessation of the statin. Therefore, the current study evaluated the protein and mRNA expression status of three known pain receptors in the testes following treatment with rosuvastatin and simvastatin.

Pain is initiated when the terminal endings of nociceptors are activated by noxious chemicals, or mechanical or thermal stimuli. The nociceptors transmit information regarding tissue damage to the pain-processing centres in the spinal cord and brain. During the transmission of pain signals, endogenous factors are released to stimulate the receptors of pain. Thereafter, these receptors are activated, and the feeling of pain ensues. According to periodic course or duration, pain can be categorized as acute or chronic. Chronic pain is further subdivided into inflammatory pain (due to activation of inflammatory cells and subsequent release of pro-inflammatory cytokines, which thereafter causes damage to the tissue or cells and, hence, pain ensues); neuropathic pain (caused by mechanical, traumatic, immunity, and tumour stimulation, which can result in motor and sensory damage, thereby inducing pain); and cancer pain (caused by tumour growth, proliferation, migration, and invasion of tumour cells, which can damage peripheral nerves, sensitize peripheral sensors, and enhance the transmission of pain information) [22,23]. Some of the known and well researched pain receptors include transient receptor potential vanilloid receptor-1 (TRPV1), purinergic receptor X2 (P2X2), and P2X3.

TRPV1, a ligand-gated, nonselective cation channel has been reported to be expressed not only in dorsal root, trigeminal, and nodose ganglia, but also in non-neuronal tissue and cells [25], including kidney, skin, intestine, heart, and testes [26]. TRPV1 can be activated by ligands (capsaicin, protons, products of arachidonic metabolism, etc.) [26,27,28,29], ambient temperature (37 degrees potentiates its responsiveness to chemical agonist, while temperatures above 42 degrees C activate TRPV1 in the absence of exogenous chemical ligand), and chemical ligands [25,52].

In non-neuronal tissues and cells, the functions of TRPV1 upon activation are cell-specific. For instance, Feng et al. reported that upon ablating TRPV1 nociceptors in mice with inflamed skin, the inflammation became more profound, and this occurred in a cause-specific manner [28]. Meanwhile, Ma et al. showed that the activation of TRPV1 by capsaicin increased cytosolic Ca^2+^ and reduced accumulation of lipids by reducing the expression of low-density lipoprotein related protein 1 and increasing ATP-binding cassette transporter A1 in the vascular smooth muscle cells. This indicates that TRPV1 activation aids cellular cholesterol efflux and reduced cholesterol uptake [53]; whereas, studies have shown that TRPV1 confers heat resistance to male germ cells [25,26]. For instance, it was shown that TRPV1 knocked out mice displayed aggressive germ cell loss, and mice experienced testicular hyperthermia [25]. These studies reiterate that TRPV1 activation in non-neuronal cells depends on the kind of noxious stimuli applied, and the response is cell- or tissue-specific.

In the current study, the testicular mRNA expression of *TRPV1* was significantly increased in the rosuvastatin-treated group, while a mild increase was observed in the simvastatin-treated group. However, the protein expression of TRPV1 was significantly reduced in the rosuvastatin group. Currently, there are no available findings on the status of testicular mRNA expression of *TRPV1* in rosuvastatin and simvastatin treatment. Nevertheless, the study of Amorim et al. associated an increase in TRPV1 and bradykinin upon simvastatin administration to the animals developing plasma extravasation and bronchi constriction [54]. These adverse effects were attenuated upon inhibiting TRPV1 and bradykinin receptors. Therefore, it was concluded that simvastatin administration caused an increase in bradykinin, which in turn promoted NO release and consequently activated TRPV1, which further led to plasma extravasation and bronchoconstriction.

In contrast, Su et al. reported that upon treatment of endothelial cells with simvastatin, there was a time-dependent increase in intracellular calcium (Ca^2+^) level. When TRPV1 was either pharmacologically inhibited or genetically disrupted, the simvastatin-induced increase in intracellular Ca^2+^ was repudiated [30]. In this case, it can be deduced that TRPV1 is required for simvastatin-mediated intracellular Ca^2+^ influx in endothelial cells, and TRPV1—Ca^2+^ signaling is important for the simvastatin-activated CaMKII-eNOS-NO pathway.

Although these studies showed opposite roles of TRPV1 in different conditions, what is common to all are that upon simvastatin administration to endothelial cells, rats and/or mice, there is an increase in TRPV1 protein expression which leads to its participation in either driving physiological function or aiding pathogenesis.

Another study showed that upon rosuvastatin administration to rats, there was a non-significant increase in the relative protein expression of TRPV1, which contrasts with the protein expression of TRPV1 in the current study. However, there was a significant increase in the mRNA TRPV1 expression upon rosuvastatin treatment. Pilutin et al. showed that upon inhibition of CYP450 aromatase (an enzyme needed for the conversion of androgen to estrogen), there was an increased immuno-intensity of TRPV1 in rat testes. The authors also made mention of the mRNA expression of TRPV1 in the testis [26]. Therefore, the findings of the current study depict an alteration in the protein and transcripts expression of TRPV1 following rosuvastatin treatment.

Other known pain receptors are the purinergic receptors. Purinergic receptors involved in pain are mostly found in the peripheral terminals where they sense ATP leakage from damaged tissues or released from inflammatory cells [55]. P2X3 is another purinergic receptor that is involved in the pathogenesis of pain. Studies have shown that upon the overexpression of P2X3, there was an increase in the release of inflammatory cytokines and the subsequent excitation of nociceptors, which promoted the development of inflammation and the perception of pain [23]. Another study has shown that the genetic deletion or pharmacological inhibition of P2X3 significantly reduced chronic pain [56]. The study of Xiang et al. showed that P2X3 was upregulated in inflammation [55]. This was supported by the study of Xia et al., who showed that increased P2X3 aided Ca^2+^ influx and caused neuronal excitability, and further led to nociception. Upon P2X3 knockdown, the chronic pain perception was reversed [56].

Although protein expression of P2X3 was not significantly increased in the current study, there was an increase in trend in the simvastatin (73.4%) and rosuvastatin (85.4%) treatment groups. Since there was a significant increase in mRNA expression of *TRPV1* and an increase in protein expression of P2X3, we investigated the expression status of inflammatory cytokines. Our findings showed that upon treatment with rosuvastatin and simvastatin, there was a significant increase in the testicular protein expression of GP130 (a transmembrane protein which is the founding member of the class of all cytokine receptors. It forms one subunit of the type I cytokine receptor within the IL-6 receptor family) and a non-significant increase in the mRNA levels of *IL-6.* Therefore, the results of the current study concur with the idea that there is a plausible association between the perception of pain and the enhancement of pro-inflammatory cytokines.

Although the direct relationship between an increase in *TRPV1* and *IL*-*6* and the perception of testicular pain was not measured, findings from this study show the possibility of developing testicular pain upon rosuvastatin administration, as the expression of these biomarkers were highest in the testes of these animals. Additionally, with the understanding that altered steroidogenesis may enhance the increased generation of pro-inflammatory cytokines [20], it can be speculated that the relationship is in fact ternary. That is, there is an association between an increase in inflammatory cytokines, altered steroidogenesis, and the expression of pain receptors.

Taken together, the influx of statins into the testicular environment is possible. However, it is still unknown whether it is the bioactive form or the metabolized form of statin that would be redistributed into the testes. Nevertheless, from the findings of the current study, statins—especially rosuvastatin—adversely affected sperm concentration, gonadal hormone receptors, and the expression of TRPV1, thereby suggesting that rosuvastatin may reduce steroidogenesis, disequilibrate the pain-inflammatory pathway, and ultimately impact male reproductive parameters negatively.

## 4. Materials and Methods

### 4.1. Ethics and Animal Care

Ethics approval was obtained from the Mohammed Bin Rashid University of Medicine and Health Sciences Ethics Committee and the Dubai Pharmacy Girls College Ethics Committee (ERA-2019-5938). Animals were treated according to the recommendations of the Laboratory Animal Care of the National Society of Medical Research and the National Institutes of Health Guide for the Care and Use of Laboratory Animals [57]. Healthy adult male Wistar rats weighing 200–250 g at start of experiment were housed in standard ventilated cages and were exposed to a 12-h light: 12-h dark cycle at 23 °C ± 2 °C in the Animal Housing Unit of the Dubai Pharmacy Girls College. Animals had free access to food and water throughout the duration of the study. Animals were acclimatized before the start of experiment.

### 4.2. Study Design

Thirty adult male Wistar rats (200–250 g) were divided into three groups of ten each. Animals were either treated orally with simvastatin (Zacor, Dubai, United Arab Emirates) (50 mg/kgbw; group 1), rosuvastatin (Crestor, Dubai, United Arab Emirates) (50 mg/kgbw; group 2), or with 0.5% carboxy methyl cellulose (Sigma Aldrich, St. Louis, MO, USA) (control; group 3), for a 30-day period. Thereafter, animals were sacrificed via sevoflurane inhalation; the testes and epididymis were collected. The retrieved testicular and epididymal tissues for protein analysis and other biochemical assays were stored at −80 °C until analysis. The testes used for Sertoli cells and Leydig cells isolation were immediately placed on ice after retrieval in an aseptic environment. Previously established protocols were followed with some modifications [58,59]. For immunofluorescence, after sevoflurane inhalation, animals were transcardially perfused with 4% paraformaldehyde in 0.1% phosphate buffer (pH 7.4) for 20 min to allow for fixation. Thereafter, testes were retrieved from the animals and placed in 10% buffered formalin for 24–48 h and continued with further processes.

### 4.3. Sperm Retrieval for Concentration and Morphology

The harvested left epididymis was defatted and placed in a petri dish containing a 2 mL solution of DMEM-Hams F-12 nutrient media (Sigma Chemicals, St Louis, MO, USA), at 37 °C. After rinsing, sperm solution for concentration and morphology analysis was obtained by dissecting the caudal area into smaller pieces, and left for 5 min allowing a maximum number of spermatozoa to swim out. The pieces were removed after 5 min, and the sperm solution was mixed until homogenous. Of the 2 mL solution, 10 µL was diluted in 50 µL DMEM-Hams. From this solution, 2 µL was infused into a chamber slide and analyzed via CASA (MicrOptic, Barcelona, Spain). The dilution was done to avoid the overlapping of cells and to allow the SCA to measure the concentration of cells accurately and efficiently in the sperm solution.

Morphology analysis was carried out in accordance with the MBRU Andrology laboratory and previously published protocols [60]. The percentage of morphologically normal spermatozoa were recorded.

### 4.4. Isolation of Leydig Cells and Sertoli Cells

The method of testicular digestion and spermatogenic cells isolation described by Chang et al. was employed in the current study with a few modifications [58]. Briefly, the tunica albuginea of the testes were removed in an aseptic environment. Thereafter, the seminiferous tubules were transferred to a 50 mL conical tube containing enzymatic solution A (DMEM-F12 (Gibco, ThermoFisher, Waltham, MA, USA), 1 mM L-glutamine (Sigma-Aldrich, USA), 5 mM sodium L-lactate (Sigma-Aldrich, USA), 1 mM sodium pyruvate (Sigma-Aldrich, USA), 0.1 mM MEM (Sigma, USA), 200 µg/mL DNASE I (Invitrogen, ThermoFisher, USA), 0.5 mg/mL collagenase IA (Gibco, ThermoFisher, USA)) for the first digestion.

### 4.5. Isolation of Leydig Cells

Tubules were incubated with enzymatic solution A at 35 °C, 80 rpm or oscillation/minute for 25 min. Thereafter, tubules were layered over 5% percoll (Sigma-Aldrich, USA) in 1X HBSS (Gibco, ThermoFisher, USA) and allowed to rest for 30 min. The top 95% of total suspension were then removed and aliquoted into another tube for the isolation of Leydig cells, while the remaining 5% of total tubule suspension was used for Sertoli cell isolation. The top 95% was washed with 1X DPBS by centrifuging at 500× *g* for 10 min at 4 °C. The supernatant was thereafter removed, and the pellet was resuspended in 55% percoll in 1X HBSS, and further centrifugation was performed at 20,000× *g* for 1 h 30 min at 4 °C. The percoll was removed, and the pellet was further resuspended in 1X HBSS and centrifuged at 500× *g* for minutes at 4 °C. After washing, the supernatant was discarded, and the pellet was resuspended in 500 µL 1X HBSS. From this Leydig cell suspension, 5 µL was added to 5 µL of trypan blue for viability assay. Retrieved Leydig cells were counted, and the percentage of viable cells was determined using a Cell Counter.

### 4.6. Isolation of Sertoli Cells

The bottom 5% of total tubule suspension after supernatant removal (95% for Leydig cell) was used for Sertoli cell isolation. To this tubule suspension fraction, enzymatic solution B (DMEM-F12, 1 mM L-glutamine, 5 mM sodium L-lactate, 1 mM sodium pyruvate, 0.1 mM MEM, 20 µg/mL DNASE I, 1 mg/mL trypsin or 0.5 mg/mL pancreatin) was added for the second enzymatic digestion. Tubules were incubated with the enzymatic solution B for 25 min at 35 °C at 80 rpm or oscillation/minute. Digestion was halted by adding 3 mL of inactivated fetal bovine serum (FBS). The suspension was filtered through a 70 µm and 40 µm cell strainers. The strainers were inverted into a separate conical tube and washed with growth media DMEM-F12, 1 mM L-glutamine, 5 mM sodium L-lactate, 1 mM sodium pyruvate, and 0.1 mM MEM. Cell viability was assessed as described above.

### 4.7. Quantification of Genes

#### RNA Isolation and Reverse Transcriptase (RT)-PCR

Total RNA was extracted from whole testis and spermatogenic cells (Sertoli and Leydig cells) using the RNeasy isolation Kit (Qiagen, Germantown, MD, USA) according to the manufacturer’s instructions. Total RNA was eluted in ~50 μL RNase-free water, and the concentration and purity were measured on Nanodrop at 260/280 nm, with a purity ratio of ~2.0. RNA Eluent was stored at −80 °C prior to use. cDNA was prepared from 1 μg of total RNA in 20 μL reaction mixture using random primers according to a standard protocol (QuantiTect Reverse Transcription Kit, Qiagen, USA). The reverse transcription reactions were performed on the T100 thermocycler (BioRad, Hercules, CA, USA), and thereafter proceeded with PCR.

qPCR was performed on 10 ng/µL of cDNA using the HOT FIREPol EvaGreen qPCR Supermix, 5x (SolisBiodyne, Tartu, Estonia) for measuring genes of interest (*SLCO1BI*, *SLCOIB2*, *SLCOIB3*, *FSHR*, *LHR*, *IL-17A*, *IL-17B*, *hGAPDH*, *GAPDH*, *IL-6*, *P2X1*, *P2X3*, and *TRPV1*). The characteristics of the primers used are shown in Table 1. Quantitative RT-PCR was performed in duplicate in a QuantStudio™ 5 System (Applied Biosystems™ A28139, Waltham, MA, USA). Data were analyzed using the QuantStudio™ Design & Analysis Software v1.4.1, and the comparative Ct method 2^−(∆Ct)^ was used to quantify gene expression levels. Data of qPCR products were standardized to GAPDH, which was used as an internal control.

### 4.8. Western Blot

Testes homogenates and protein determination were obtained as previously described [60]. Lysates were prepared by diluting samples in Laemmli sample buffer and lysis buffer, boiled for 5 min, and thereafter separated 50 µg protein/µL by electrophoresis on a 12% SDS-PAGE mini-proteon gel. Proteins were transferred onto a Millipore Immobilon-P transfer membrane (0.45 µm) (Immobilon^®^-P, Merck Millipore Ltd., Darmstadt, Germany). Non-specific sites were blocked with 5% fat-free milk in TBS-Tween. Primary antibodies were diluted in TBS-Tween in a biomarker dependent concentration, while the secondary antibodies were diluted in TBS-Tween in a 1:4000 ratio. The proteins measured were TRPV1, P2X1, P2X3, LHR, FSHR, and GP130.

### 4.9. Immunofluorescence

After isoflurane inhalation, animals were perfused with 4% paraformaldehyde for 20 min to allow for fixation. Thereafter, testes were retrieved from the animals and placed in 10% buffered paraformaldehyde for 24–48 h, and thereafter followed previously described methods for clearing, embedding, and sectioning [61]. Sections were deparaffinized at 58 °C for 45 min and rehydrated in graded alcohol for 5 min each. Thereafter, they were washed in tap water for 3 min and then placed in antigen retrieval solution (citrate buffer pH 6.0) for 30 min at 95 °C. Sections were cooled to room temperature and then washed in TBS for 15 min, repeated 3 times, and thereafter probed with freshly prepared 3% H_2_O_2_ for 5 min, and thereafter washed in TBS for 15 min at 3 intervals. They were then probed with primary antibodies of TRPV1, P2X1, P2X3, LHR, FSHR, and GP130 overnight, and thereafter probed with Alexa Fluor 488 secondary antibody and counter stained with DAPI mounting media. Micrographs were taken with a fluorescence microscope (Olympus BX41). All primary antibodies were procured from Alomone labs (Jerusalem, Israel), while the secondary antibody was obtained from Abcam (Cambridge, UK).

### 4.10. Statistics

GraphPad Prism™ software (GraphPad™ Software, Version 9.3.1, San Diego, CA, USA) was used for the statistical analysis. The current study was restricted to a sample size of 10 per group because of the 3Rs (replace, reduce, refine) imposed for animal use. Additionally, with statistical power accepted at 80% (0.8), and type I error at 0.05 (5%), the sample size required per group is 10. Normal data distribution was measured using the Shapiro-Wilk, Anderson-Darling, Kolmogorov-Smirnov, and D’Agostino and Pearson normality tests. When data passed all normality tests, a one-way ANOVA of variance with a Tukey’s Post hoc Test was performed. Where data were not evenly distributed, a Kruskal-Wallis test and a Dunns Post hoc Test were carried out. A probability level of *p* < 0.05 was considered statistically significant, and results were expressed as mean ± SD.

## 5. Conclusions

Statins indeed remain the gold standard therapy for hypercholesterolemia. However, many side effects are still being reported. Although the influence of statins on fertility is not widely investigated, several studies have demonstrated their effects on male reproductive parameters and health. Concerns about the possibility of causing testicular pain have also been raised. Hence, the current study investigated the effect of simvastatin and rosuvastatin on male reproductive parameters, and examined pathways through which the adverse effects are exerted. Findings showed that rosuvastatin caused reduced sperm concentration, while this effect was not seen in simvastatin-treated animals. Furthermore, findings showed the presence of *SLCO1B1*, *SLCO1B2*, and *SLCO1B3* in the Sertoli cells, Leydig cells, and whole testicular homogenate. Additionally, there was an increase in the mRNA levels of *TRPV1*, IL-*6*, and elevated protein expression of GP130. Reduced LHR and FSHR protein expression was also seen. Since there are no available data on whether the transporters of statins and the genes that encode the function of these transporters are present in the whole testes and/or in the diverse spermatogenic cells, the evaluation of the expression status of these genes sheds more insight into understanding the underlying pathogenesis of statin-related testicular discomfort and male infertility.

Taken together, the adverse effect of rosuvastatin, in particular, on male reproductive parameters may be exerted through the downregulation of gonadal hormone receptors, the altered expression of pain channel receptors, and elevated inflammatory cytokines. Findings from the current study explored the possibility of statin-induced testicular pain, an idea that requires further investigation. These findings, if confirmed in clinical studies, may help clinicians to make informed decisions, especially when attending to men who still have the desire to father children, or men who are already seeking infertility treatment.

### Study Limitation

We did not identify the presence and or activity of these transporters (OATPs) directly on the blood-testis-barrier (BTB), which may inform us of the permeability of statin from the systemic circulation into the testicular microenvironment. However, the study of Hau et al. already established the presence of these transporters on the BTB; hence, we built on that study. Another flaw is that we did not investigate the functionality of the transporters in the cells where expression was found, due to the age of the animals used. For this study to investigate the effect of the statins studied on male reproductive parameters, adult male Wistar rats are needed. However, functionality assays can only be performed on proliferating Sertoli cells, which can only be obtained from younger animals. Therefore, future studies may culture Sertoli cells isolated from 6–12 days old rats to investigate the role of statin during BTB formation, investigate the functionality status of these transporters, and corroborate their genetic expression to their activity. Finally, future studies may examine the presence and expression of these transporters on all spermatogenic cells, as this will inform us of the capability of the activation or deactivation of the transporters on each phase of spermatogenesis.

## Figures and Tables

**Table 1:** Characteristics of the primers for the genes of interest.

Genes	Forward	Reverse	Amplicon Size
*TRPV1*	TCCAAGGCACTTGCTCCATT	TGGAGGTGGCTTGCAGTTAG	181
*P2X1*	CGGATGGTGCTGGTACGAAA	CTTCACGGACACACTGCTGA	147
*FSHR*	CTGTACATCAACCCGGAGGC	TGAAGGAGTTCCTGGCAACG	172
*LHR*	GCCTCGCCAGACTATCTCT	TTGAGGAGGTTGTCAAAGGC	148
*P2X3*	AGGACATAAAGAGGTGCCGC	GGCCTTGTCTAGATCGCACA	164
*IL-6*	CACTTCACAAGTCGGAGGCT	TCTGACAGTGCATCATCGCT	114
*SLCO1B2*	GAGCTCAGGGTAGACGACCA	TGTAGCTGAAGGAGAGGGCT	197
*IL-17A*	AGCTGATCAGGACGAGCGAC	GGGATGAGTACCGCTGCCTT	110
*GAPDH*	CCGCATCTTCTTGTGCAGTG	CGATACGGCCAAATCCGTTC	79
*GAPDH-H*	AGGTCGGAGTCAACGGATTT	TGACAAGCTTCCCGTTCTCA	192
*SLCO1B1-H*	AAAGGGTGGACTTGTTGCAG	GTGACAGAGCTGCCAAGAAC	210
*SLCO1B3-H*	CGTTGTTCCCACCTCCTAGA	TGAATCCATTGCAGCGTCTT	196
*IL-17B-H*	ATTCTCTCGGCGGCATCTG	CCTTCCTCTTGCTTTTGGGG	158

Unless otherwise stated, primers are from Rattus navergicus. Suffix ‘H’ = Homo sapiens.

## Data Availability

Data are contained within the article.
